# Retreatment of patients with chronic hepatitis C, subtype 3a, and cirrhosis, who previously failed a regimen containing second-generation NS5A inhibitors with sofosbuvir + glecaprevir/pibrentasvir and ribavirin for 16–24 weeks

**DOI:** 10.1128/jvi.01843-24

**Published:** 2025-01-22

**Authors:** Sergii V. Fedorchenko, Zhanna Klimenko, Tatiana Martynovich, Iryna Solianyk, Tatiana Suprunenko

**Affiliations:** 1 Department of Viral Hepatitis and AIDS, The L.V. Gromashevskyi Institute of Epidemiology and Infectious Disease 675348 , Kyiv, Ukraine; St. Jude Children's Research Hospital, Memphis, Tennessee, USA

**Keywords:** hepatitis C virus, antiviral agents, mutation, resistance

## Abstract

The outcomes of retreatment patients infected with hepatitis C virus genotype 3, cirrhosis, with velpatasvir may be affected by treatment failure with velpatasvir. The efficacy of SOF+GLE/PIB+RIB 16–24 weeks of treatment has been shown. The presence of NS5A resistance-associated substitution mutations, including Y93H, and the number and regimens of the past failed therapy do not influence the likelihood of achieving sustained virological response. When velpatasvir treatment fails, pibrentasvir should be used as the first choice for retreatment.

## COMMENTARY

The pan-genotypic direct-acting antivirals (DAAs) are highly effective with cure rates of generally >95%. Factors associated with treatment failure include prior treatment experience, poor adherence to treatment, presence of cirrhosis, and preexisting resistance-associated substitutions (RASs), or polymorphisms linked to certain subtypes which confer lower sensitivity to the existing DAAs.

Genotype 3 (GT3) is more prevalent in South (India and Pakistan) and Southeast Asia (Malaysia and Philippines) and can be found in more than 30% of the hepatitis C virus (HCV) population in certain European countries, such as the United Kingdom, Denmark, and Finland (1). Subtype 3a is predominant in North and South America, Australia, Europe, the Middle East, and Oceania (2), and subtype 3b—in China and Japan (3). Among Ukrainian patients with chronic hepatitis C, 30%–35% are infected with HCV subtype 3a, and 12% of those have cirrhosis (4).

GT3 is considered the most poorly understood genotype. Compared with other genotypes, GT3 infection has been associated with an increased risk of accelerated hepatic fibrosis progression (5), hepatic steatosis (6), and the development of hepatocellular carcinoma (HCC) in cirrhotic patients (7).

According to the results of national studies in several European countries, the efficacy of the second-generation DAA therapy in naïve patients without cirrhosis infected with HCV GT3 is 96%–98% (8, 9), and in those with cirrhosis, 93%–97% (9–11). About 3%–5% of patients treated with second-generation DAAs develop virological breakthrough or relapse (11–13). In patients who have received NS5A inhibitors and NS5B polymerase inhibitors and have not achieved sustained virological response (SVR), nucleotide substitutions in the nonstructural HCV RNA region, defined as RASs, are found in 80%–90% of cases. The presence of RAS mutations in NS5A region leads to reduced HCV sensitivity to DAAs and necessitates retreatment with antiviral therapy to achieve SVR, unlike NS5B RASs which are not stable and have less clinical significance (14–16).

We evaluated the efficacy of antiviral therapy using 400-mg sofosbuvir (SOF) + 300-mg glecaprevir (GLE)/120-mg pibrentasvir (PIB) (three pills) + 1,000- to 1,200-mg ribavirin (RBV) per day (QD) divided into two doses for 16–24 weeks in patients with HCV infection subtype 3a, cirrhosis, who had failed treatment with second-generation NS5A inhibitors (velpatasvir [VEL]). A total of 12 patients were included in the study. All the subjects had received retreatment with SOF + GLE/PIB + RBV, completed the prescribed treatment, and were evaluated 12 weeks after its completion (SVR_12_). All these patients were of Slavonic origin. There were nine men (75%) and three women (25%); the median age was 51.3 ± 10.9 years. All the patients (100%) had cirrhosis (two patients had clinical signs of decompensation in the past). Baseline high viremia >6 log IU/ml was found in four patients. In most patients, the average HCV RNA level varied within 10^3—^10^6^ IU/mL. Due to the development of HCC during antiviral therapy, one patient was excluded from further study.

Seven of 11 patients (63.6%) had one line of treatment with the first-generation DAAs (NS5A and NS5B inhibitors): SOF/DAC, five patients for 24 weeks, and SOF/DAC + RBV, two patients for 12 weeks. Four of 11 patients (36.4%) failed after the first-line treatment with the second-generation DAAs—SOF/VEL (12 weeks). After the completion of antiviral therapy, a relapse was diagnosed in nine patients and a virological breakthrough in two. The second course of treatment included SOF/VEL + RIB for 24 weeks (17). The virological breakthrough occurred in one patient, and 10 patients relapsed. The third course of treatment included SOF + GLE/PIB + RIB for 16–24 weeks. For three patients with an initial high level of HCV RNA viremia (>6 log IU/ml) and a double NS5A mutation, we recommended a 24-week course of treatment.

Three of 11 patients were tested for the presence of NS5A RAS mutations before and after the second course of antiviral therapy with SOF/VEL + RIB for 24, which failed. Before the start of retreatment, the Y93H mutation was detected in all of them; after the relapse was diagnosed, the Y93H mutation continued to be detected in two patients, and in one more, in addition to the Y93H mutation, the A30K mutation began to be detected (17). Assessment of the resistance profile prior to initiation of retreatment with SOF + GLE/PIB + RBV has revealed that major mutations were localized in NS5A region.

RAS mutations in the NS5A region were identified in all 11 patients. The most frequently found mutation was Y93H, which was detected in nine out of 11 patients (81.8%), followed by L31M/I/V in seven (63.6%), and finally A30K in two (18.2%). Of note was a significant number of patients with two mutations—seven out of 11 patients (63.6%). Thus, the L31M/I/V + Y93H combination was determined in five persons (45.5%) and A30K + Y93H in 2 (18.2%). Single mutations were found in four persons (36.4%), Y93H, two (18.2%), and L31M/V, two (18.2%).

RASs in the NS5В region were detected in two persons (18.2%) S282T. Those were the patients who had failed treatment with the SOF/VEL + RIB for 24 weeks 3 and 2 months before retreatment with SOF + GLE/PIB + RIB.

Of 11 patients who started therapy, eight individuals (72.7%) achieved rapid virologic response (RVR) on week 4 and 11 (100%), SVR_12_. None of the mentioned baseline factors alone impacted significantly the efficacy of retreatment.

Selecting retreatment with the second-generation DAAs for patients who had failed treatment with NS5А inhibitors (second-generation) is very complicated. There exist three main rules of retreatment: use of drugs from other classes that do not have cross-resistance with the drugs that have been already used (18), prolonging the earlier treatment regimens with or without the addition of RBV (19, 20). Following these rules in retreatment of patients with HCV GT3a infection results in the achievement of SVR in 88%–100% (21–23).

Current protocols mandate retreatment with GLE/PIB and SOF/VEL/voxilaprevir in patients who have failed treatment with NS5A inhibitors. However, these recommendations are difficult to implement for at least two reasons.

Firstly, patients with cirrhosis are the most difficult target for retreatment especially if pangenotypic regimens of the second-generation DAAs (GLE/PIB, SOF/VEL/ voxilaprevir) are not registered in the country, or they are not reimbursed by the state healthcare system. Secondly, some patients with compensated cirrhosis (Child–Pugh class A) may have had clinical signs of decompensation in the past. Among our 11 patients, a history of clinical signs of decompensation was recorded in two patients (two had ascites). According to current treatment protocols, the use of protease inhibitors (GLE and voxilaprevir) is not recommended in this category of patients (19).

Until October 2019, we were not able to retreat patients with HCV subtype 3a, cirrhosis, who had failed treatment with second-generation NS5A inhibitors using GLE/PIB or SOF/VEL/VOX. All patients have been treated with SOF/VEL + RIB for 24 weeks. According to our previously published data, the treatment efficacy was 83.3% (40/48 patients) and varied depending on subgroups. In the group of patients with the presence of RAS NS5A, A30K or L31M, SVR was observed in 87%, in the presence of the Y93H mutation—in 78%. The lowest rates of SVR induction were recorded in the group of patients with the presence of RAS NS5A, Y93H, who had failed treatment with second-generation NS5A inhibitors—73% (VEL) (17). Patients who failed after treatment with SOF/VEL + RIB for 24 weeks were retreated with SOF + GLE/PIB + RIB for 16–24 weeks.

The S282T mutation in RASs in the NS5B region increases SOF EC_50_ by only fourfold, is rare, and has no significant clinical significance.

Baseline NS5A RAS in naïve patients infected with HCV subtype 3a occurs in Europe in 8.9%–15.8% and varies widely by region (23, 24). The most clinically significant mutations in NS5A are A30K, L31M, and Y93H (24–26). Antiviral therapy with SOF/VEL ± RIB provided for 12–24 weeks or GLE/PIB for 8–16 weeks for naïve patients with and without compensated cirrhosis led to the achievement of comparable SVR induction rates 95.7% and 96.7% (8), regardless of the presence of baseline NS5A RAS (10–12, 27, 28). Virological breakthrough or relapse was diagnosed in 2%–4% of patients (29, 30).

These very emerging mutations are most often registered, with more than 80% resistance, in patients with HCV subtype 3a in case of failure of the most commonly used second-generation DAA treatment regimens: SOF/VEL and GLE/PIB (31–33). As mentioned above, the most common cause of failures of DAA treatment are RASs in the NS5А region, with A30K, L31M, and Y93H being the most significant of them. In patients infected with HCV subtype 3a, the substitution of Y93H in NS5A increases VEL EC_50_ >200-fold, but PIB only EC_50_ >2- to 4-fold (15, 34). Double mutation A30K + Y93H reduces PIB EC_50_ >50-fold, the single one A30K only EC_50_ >1.1-fold (34).

According to our data, NS5A RASs were registered in 11 (100%) out of 11 patients infected with HCV subtype 3a.

The impact of NS5A RASs on the achievement of SVR _12_ was evaluated in patients with the presence of A30K, L31M, Y93H, and double mutations.

As a result of retreatment with SOF + GLE/PIB + RIB for 16–24 weeks, HCV RNA was not detected in eight (72.7%) patients at 4 weeks of treatment (RVR); all patients achieved SVR, regardless of the previous unsuccessful treatment regimens, baseline level of viremia, and the presence of single or double mutations of RAS NS5A. The clinical trial MAGELLAN-3 demonstrated the high effectiveness of the retreatment regimen SOF + GLE/PIB + RIB for 16 weeks in a group of patients with HCV GT3 infection who had failed treatment with second-generation NS5A inhibitors (PIB) and the presence of NS5A RASs. As a result of the treatment of 14 patients, all of them achieved SVR (21). The high treatment efficacy has been confirmed in a number of other studies, both in patients with and without cirrhosis, in the presence of infection with HCV subtypes 3a and 3b, after the failure of the first course of treatment—GLE/PIB (22). PIB is a second-generation NS5A inhibitor with a very high resistance threshold, especially to the most common mutation—Y93H. Therefore, in the process of re-therapy, often the second-generation NS5A inhibitor (PIB) is not changed, using two other basic rules of retreatment out of three: prolonged therapy and the addition of ribavirin (22).

At this stage of the presentation of the material, it is important to address the issue of the need to add ribavirin to the retreatment regimens. In naïve patients without cirrhosis, the addition of ribavirin to SOF + second-generation NS5A inhibitor (VEL) regimen has increased the likelihood of achieving SVR by an average of 2%–3% compared with patients receiving SOF + second-generation NS5A inhibitor alone (VEL) (35). In the presence of compensated cirrhosis, the difference in SVR achievement could be 5%–7% in favor of patients receiving ribavirin. However, these differences were also found to be insignificant (36, 37). Further studies have demonstrated that an increase in SVR induction in the group of naïve patients receiving additional ribavirin is associated with greater efficacy of SOF + second-generation NS5A inhibitor (VEL) + RBV in patients with baseline NS5A RASs, especially those with the Y93H mutation (37, 38). Given the frequency of baseline NS5A RASs in naïve patients (8.9%–15.8%) and the emergence of NS5A RASs in patients who fail treatment with NS5A inhibitors (80%–90%), it is clear why current retreatment regimens recommend the addition of ribavirin (19, 20). It should be noted that the above is more relevant to retreatment regimens that include SOF/VEL ± RIB, since the results of retreatment with SOF/VEL/VOX ± RIB and SOF + GLE/PIB ± RIB are less dependent on the addition of RIB (39, 40).

In recent years, a number of studies have appeared on the efficacy of 16- to 24-week regimens of SOF + GLE/PIB + RIB in a group of patients infected with HCV GT3 who had failed treatment with SOF/VEL/VOX. In one report, all patients with HCV GT3 infection, with and without cirrhosis, with key NS5A RAS mutations (L31M, A30K, and Y93H) achieved SVR on a 16- to 24-week regimen (41). In another study, 18 patients with HCV GT3 who had failed treatment with SOF/VEL/VOX were retreated with SOF + GLE/PIB + RIB, GLE/PIB, SOF/VEL + RIB, and SOF/VEL/VOX + RIB. Only one patient treated with SOF + GLE/PIB + RIB had documented relapse; all others achieved SVR (42).

When discussing options for retreatment, one cannot help but touch upon the issue of the efficacy and safety of SOF/VEL/VOX in patients with GT3 who had failed treatment with second-generation inhibitors. The ASTRAL-3 study showed that the efficacy of SOF/VEL therapy for 12 weeks was highly dependent on the presence of the underlying NS5A RASs, particularly Y93H. Thus, in the absence of the Y93H mutation, SVR was achieved in 97.4% of patients, and in the presence of NS5A RASs—in 88.4%, the lowest SVR rates were recorded in the presence of the Y93H mutation—84%. The presence of cirrhosis was a negative predictive factor (9). The POLARIS-4 study assessed the efficacy of retreatment with SOF/VEL/VOX in GT3 patients with experience of treatment with NS53 and NS5B inhibitors, but not with NS5A (43). Therefore, the influence of NS5A RASs, the grade of fibrosis, and other factors were assessed based on the results of the actual clinical practice. Initial clinical reports indicated 100% efficacy and absence of effect on SVR of baseline RASs, prior therapy regimens, grade of fibrosis, or baseline level of viremia (44, 45). In the course of accumulating clinical material and assessing the influence of predictive factors on SVR achievement, it became clear that past exposure to SOF/VEL and/or complex resistance profiles, past liver transplantation, and the presence of HCC and cirrhosis are the main negative predictors of achieving SVR (46, 47). Retreatment with SOF/VEL/VOX in patients with HCV GT3 led to the achievement of SVR only in 81%, in the presence of cirrhosis, in 81%, with the lowest SVR rates noted in the group of patients who had failed SOF/VEL treatment, 71% (46). Taking into account the low rates of SVR induction, the group of authors analyzed more than 740 cases of SOF/VEL/VOX retreatment failure in six European countries: Austria, Belgium, Germany, Italy, Spain, and Switzerland. The authors concluded that the main negative predictive factors included HCV GT3, presence of HCC, cirrhosis, and past SOF/VEL therapy. As salvage therapy, the authors recommend SOF + GLE/PIB ± RIB for 12–24 weeks (48).

This is a prospective study with RAS data for all, while including *n* = 11 participants is potentially actually the largest sample size of GT3a treatment failures, with cirrhosis, who have been retreated with SOF + GLE/PIB + RBV. Retreatment was highly effective and safe in patients with chronic HCV GT3a infection, including those with liver cirrhosis, who previously failed DAA containing second-generation NS5A inhibitors. When VEL treatment fails, PIB should be used as the first choice for retreatment.
